# Retinopathy of prematurity and risk factors: a prospective cohort study

**DOI:** 10.1186/1471-2431-5-18

**Published:** 2005-06-28

**Authors:** Padmani Karna, Jyotsna Muttineni, Linda Angell, Wilfried Karmaus

**Affiliations:** 1 Division of Neonatology, Pediatrics and Human Development, Michigan State University, East Lansing, USA; 2 Department of Epidemiology, College of Human Medicine, Michigan State University, East Lansing, USA

## Abstract

**Background:**

Increased survival of extremely low birth infants due to advances in antenatal and neonatal care has resulted in a population of infants at high risk of developing retinopathy of prematurity (ROP). Therapeutic interventions include the use of antenatal and postnatal steroids however, their effects on the severity of ROP is in dispute. In addition, it has not been investigated whether severe ROP is due to therapeutic interventions or due to the severity of illness. The aim of the present study was to assess the association between the incidence of severe retinopathy of prematurity (greater than stage 2 – International classification of ROP) and mechanical ventilation, oxygen therapy, gestational age, antenatal and postnatal steroids in extremely low birth weight infants.

**Methods:**

Neonates admitted to the neonatal intensive care unit in Lansing, Michigan, during 1993–2000 were followed to determine factors influencing the development of severe retinopathy of prematurity. Ophthalmologic examinations were started at 6 weeks and followed until resolution. We used logistic regression to estimate the relative risk (odds ratio) associated with risk factors of ROP.

**Results:**

Of the neonates with ≤ 1500 g birth weight, admitted to the neonatal intensive care unit, 85% (616/725) survived. Severe retinopathy of prematurity was detected in 7.8% of 576 neonates who had eye examinations. Neonates of lower gestational age (≤ 25 weeks and 26–28 weeks) had an increased odds ratio of 8.49 and 3.19 for the development of severe retinopathy of prematurity, respectively, compared to those 29 weeks and older. Late postnatal steroid treatment starting after 3 weeks of life showed 2.9-fold increased odds ratio, in particular administration for two weeks and more (OR: 4.09, 95% CI: 1.52–11.03). With increasing antenatal steroids courses the risk of severe retinopathy of prematurity decreased, however, it was not significant. Lower gestational age, dependence on ventilation, and use of postnatal steroids were intertwined. Simultaneous presence of these factors seems to indicate severe disease status.

**Conclusion:**

Prolonged and late postnatal steroids treatment in very low birth weight infants may pose an increased risk for the development of severe retinopathy of prematurity; however, use of postnatal steroids may also be a marker for severity of illness. Further studies need to focus on biologic markers in the pathogenesis of retinopathy of prematurity and to better understand the influence of therapies.

## Background

Retinopathy of prematurity (ROP) is the main cause of visual impairment in premature infants [1]. The increased survival of extremely low birth weight (ELBW) infants in recent years, due to advances in neonatal care, has produced a population of infants at very high risk of developing ROP [2]. It has been believed for many years that oxygen therapy increases the risk of ROP in preterm infants [3]. However, ROP can occur even with careful control of oxygen therapy [4]. Several factors increase the risk of ROP, especially those associated with short gestation and low birth weight [5]. Other identified risk factors include sepsis, intraventricular hemorrhage, exposure to light [6], and blood transfusions [7], and mechanical ventilation [8].

One of the recent changes in the care of low birth weight (LBW) infants is administration of antenatal steroids (ANS) to reduce the risk of respiratory distress syndrome (RDS) and neonatal death in preterm neonates [9]. The 1994 National Institute of Health consensus development conference recommended the administration of ANS for pregnancies 24 to 34 weeks threatened with premature delivery [10]. Despite strong support for giving a single course of ANS, the need is far less clear for continuing this regimen in high-risk women who have not delivered one week after their initial course. A survey of members of the Society of Perinatal Obstetricians in 1995 reported that 96% of respondents were willing to give more than one course of ANS and 58% would give more than six courses of ANS [11]. In addition, postnatal steroids (PNS) have been used increasingly for the prevention and treatment of Chronic Lung Disease (CLD) in LBW infants [12]. However, reports concerning the association between postnatal dexamethasone (PNS) use and the incidence of ROP are contradictory [13-16]. Recent Cochrane reviews concluded no significant effect of early therapy with PNS on severe ROP, but late PNS use revealed increased risk of severe ROP (RR = 1.52, 95%CI: 1.09–2.12) [17-19]. However, the conclusion for late PNS use is based only on 241 children from six studies.

Not only are there controversies around the effect of antenatal and postnatal steroids on the incidence and severity of ROP in ELBW infants, effects of multiple antenatal steroid courses on the incidence and severity of ROP has not yet been assessed. The primary focus of this study was to examine the relationship between severe ROP and ANS courses, late use of postnatal steroids and its duration, mechanical ventilation, oxygen therapy, and gestational age.

## Methods

### Subjects and clinical data

All very low birth weight (VLBW, ≤1500 g) neonates, hospitalized in the Neonatal Intensive Care Unit (NICU) at Sparrow Hospital from January 1993 to July 2000 and who survived until discharge, were eligible for the study. Infants with lethal congenital anomalies were excluded. Data for all patients admitted to the NICU were entered into a computerized neonatal database (medical data systems, Pennsylvania). We retrieved and analyzed data for these patients. Antenatal steroids (Betamethasone) 12 mg, 2 doses at 24-hour intervals were administered prenatally to women with threatened preterm labor at 24 – 34 weeks gestation. Gestational age was determined by either the last menstrual period or ultrasound and confirmed by neonatal examination. Surfactant was given to infants who met clinical and radiologic criteria for respiratory distress syndrome (RDS) as a rescue treatment within 2–6 hours of life. Patent ductus arteriosus was diagnosed by echocardiography and treated per protocol. Intraventricular hemorrhage was diagnosed by serial cranial ultrasound studies. Chronic Lung Disease (CLD) was defined as a requirement for supplemental oxygen at 36 weeks post-conceptional age and chest x-ray changes consistent with CLD. Usually 3–4 weeks after delivery, postnatal dexamethasone treatment was initiated at the discretion of the individual neonatologist for the treatment of CLD, for high oxygen need, or inability to wean infant from respiratory support. A single pediatric ophthalmologist did all eye examinations starting at 6 weeks after delivery. Follow up examinations were done until the resolution of ROP or retinal maturation and used the international classification of ROP to classify the severity [20]. None of the babies received additional supplementation of Vitamin E.

### Statistical analyses

In order to determine whether the incidence of ROP was increased in specific exposure levels, we grouped continuous variables: gestational age: ≤ 25 weeks, 26–28 weeks, and > 28 weeks; exposure to ANS: no, 0.5, 1, 2, ≥ 3 courses; duration of ventilation: no, 1, 2, ≥ 3 weeks, exposure to PNS: no, 1, 2, ≥ 3 weeks. To assess the relative risk of PNS in multivariate models, we decided to dichotomize post-natal steroids use into any exposure versus no exposure.

We assessed risk factors for severe ROP (>stage 2). Since we had twins in the sample and data of twins are correlated, the use of logistic regression would not be justified. Hence we used Generalized Estimation Equations (SAS, PROC GENMOD) that allows adjusting for autocorrelation between observations [21]. We estimated odds ratios (OR) and their 95% confidence intervals (95% CI) for all potential risk factors. We then reduced the model to the most parsimonious set of predictors, by eliminating all potential predictors that showed no important association (OR not different from 1) and that did not change the OR of the other predictors by more than 10% when eliminated from a full model. All above mentioned procedures were carried out using SAS software version 8.02 [22].

The study protocol was approved by the human subject review committee of Michigan State University.

## Results

**Figure 1:**
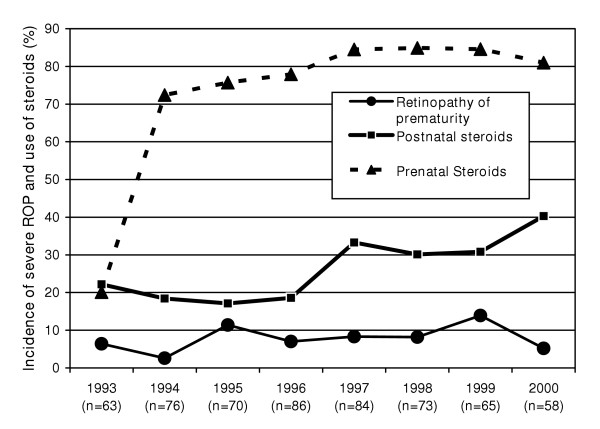
Incidence of severe retinopathy of prematurity (> stage 2 – International classification of ROP) and treatment with prenatal and postnatal steroids in the neonatal intensive care unit, Lansing, Michigan.

During the observation period from 1993 to 2000, 725 very low birth weight (VLBW) infants were admitted to the neonatal intensive care unit (NICU). A total of 616 infants (85%) survived until hospital discharge. Eye examination was performed on 576 infants; 40 infants (6.5%) were lost to follow up. From 1993 to 2000, the incidence of severe retinopathy of prematurity (ROP) changed only slightly in our neonatal intensive care unit (Figure 1). The use of antenatal steroids showed a major increase during 1993 – 1994 and a minor change thereafter. The use of PNS increased during the last four years of the study. Hence on this aggregative level, there seems to be no association between severe ROP with antenatal or postnatal use of steroids.

However, on an individual level the incidence of severe ROP was associated with postnatal administration of steroids for greater than 1-week duration (Table 1). Beyond that, there was an obvious increase with gestational age and a reduced incidence in African American neonates. The explanatory model for severe ROP, mutually controlling for all predictors, is provided in Table 2. Compared to Caucasian children, the odds ratio was significantly lower in African American children. Gender, respiratory distress syndrome, oxygen treatment at 36 weeks post-conceptional age, and prenatal use of steroids were not significantly associated with severe ROP. A more parsimonious model included only three significant factors: gestational age, duration of ventilation, and postnatal steroids. The incidence of severe ROP clearly decreased with increasing gestational age (Table 2). Late postnatal treatment with steroids showed a 2.9-fold increased odds ratio (95% CI: 1.17–7.28) for the development of severe ROP. Duration of ventilation was not significant but needed to be controlled for as it changed the effect of postnatal steroids and gestational age.

**Table 1:** Incidence of Retinopathy of Prematurity (ROP) by patient characteristics.

		n	No ROP (%)	≤ stage 2 ROP (%)	> stage 2 ROP (%)
Total cohort		576	63.4	28.8	7.8
Gender	boys	308	64.6	27.0	8.4
	girls	268	61.9	31.0	7.1
Gestational age	≤ 25 weeks	61	4.9	59.0	36.1
	26–28 weeks	198	42.9	47.0	10.1
	> 28 weeks	317	87.4	11.7	1.0
Race	White	433	62.8	27.7	9.5
	Black	109	68.8	18.7	2.8
	Other	34	52.9	44.1	2.9
Use of antenatal steroids	no	153	60.1	31.4	8.5
	0.5 course	83	59.0	28.9	12.1
	1 course	236	66.1	27.5	6.4
	2 courses	51	62.8	25.5	11.8
	≥ 3 courses	53	67.9	30.2	1.9
Respiratory distress syndrome	no	293	78.5	18.1	3.4
	yes	283	47.7	39.9	12.4
Ventilator	no	200	87	12.5	0.5
	1 week	174	82.8	15.5	1.7
	>1 – 2 weeks	48	54.2	39.6	6.3
	>2 weeks	154	13.6	61.7	24.7
Use of postnatal steroids	no	422	78.7	19.2	2.1
	1 week	47	37.2	61.7	2.1
	> 1 – 2 weeks	38	15.8	52.6	31.6
	> 2 weeks	62	6.5	21.7	48.9
Oxygen for 36 weeks	no	474	70.9	23.3	4.9
	yes	102	28.4	50.0	21.6

**Table 2:** Odds ratio for risk factors of Retinopathy of Prematurity (ROP).

		Full Model		Reduced Model	
		n = 570		n = 570	
		Odds-Ratio	95% CI	Odds-Ratio	95% CI

Gestational age	>28 weeks >28 weeks	1	-	1	
	26–28 weeks	4.12	1.05–16.11	3.19	0.81–12.55
	≤ 25 weeks	11.27	2.61–48.66	8.49	2.0–35.94
Race	Caucasian	1	-		
	Other	0.22	0.03–2.0		
	African American	0.18	0.05–0.68		
Gender	Boys	1	-		
	Girls	0.71	0.33–1.52		
Antenatal steroids	No	1	-		
	0.5 course	0.78	0.27–2.28		
	1 course	0.50	0.17–1.26		
	2 courses	0.52	0.14–1.91		
	≥ 3 courses	0.17	0.02–1.49		
Respiratory distress syndrome	No	1	-		
	Yes	0.96	0.39–2.37		
Ventilation	No	1	-	1	
	1 week	2.15	0.2–22.58	2.3	0.23–23.35
	1 – 2 weeks	3.45	0.30–39.91	4.57	0.40–51.63
	> 2 weeks	8.27	0.86–79.74	9.02	0.96–85.0
Postnatal steroids	No	1	-	1	
	Yes	2.69	1.05–6.89	2.91	1.17–7.28
Oxygen for 36 weeks	No	1	-		
	Yes	1.29	0.59–2.87		

At the onset of the analysis, we dichotomized postnatal steroid application (none vs. any, Table 2). However, descriptive data in Table 1 suggested a threshold between one and two weeks of postnatal steroids used. Thus, we additionally analyzed a three-category variable. The analyses revealed that, compared to no postnatal steroid administration, there was no increased relative risk (odds ratio) for postnatal steroids used for seven days and less, but an increased risk if used for two weeks and more (OR: 4.09, 95% CI: 1.52–11.03). Additionally, we had information on the dose of postnatal steroids for 488 of 576 children. A steroid administration of two weeks or more corresponded to a dose of ≥ 7 mg in 73.7% of the neonates.

**Figure 2:**
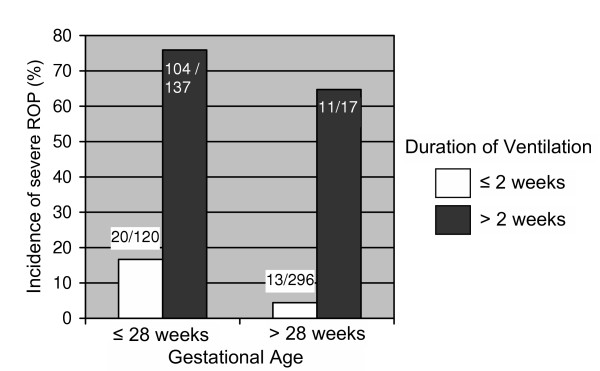
Association between gestational age, duration of ventilation and postnatal steroids administration.

**Figure 3:**
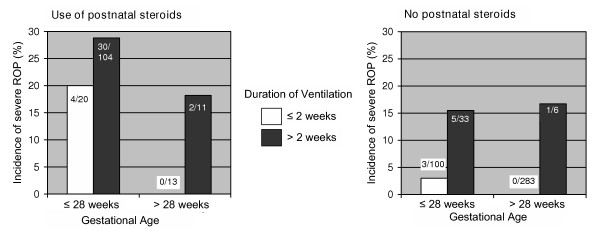
Association between gestational age, duration of ventilation, and severe ROP among VLBW infants stratified for administration of postnatal steroids.

The three factors, gestational age, duration of ventilation, and late administration of postnatal steroids, were intertwined (Figure 2). Among infants who received postnatal steroids, 65–76% were dependent on ventilation for more than two weeks, but only 4.4% (13/296) of the infants older than 28 weeks with ≤ 2 weeks of ventilation received postnatal steroids. We further attempted to test the significance of combined effects of the three predictors on the incidence of severe ROP. However, high collinearity between them averted further statistical assessments. Nevertheless, when comparing the incidence of severe ROP in different combinations of these three factors, it was notably higher in infants treated with postnatal steroids (Figure 3), in particular infants with a low gestational age and infants dependent on ventilation for more than two weeks. The majority of cases of severe ROP were in infants with lower gestational age (<28 weeks). In this group without the use of postnatal steroids, the incidence of severe ROP increased from 3% (3/100) to 15.2% (5/33); and in the group with postnatal steroids from 20% (4/20) to 28.8% (30/104) with varying duration of ventilation.

## Discussion

Our study was comprised of 576 neonates with ≤ 1500 g birth weights, of which 7.8% developed severe ROP (greater than stage 2 – International classification of ROP). Gestational age, duration of ventilation, and late postnatal steroids administration were significantly associated with severity of ROP. Gestational age and duration on ventilation were associated with development of ROP [8,23]. Consistent with published data, incidence of severe ROP was higher in Caucasians than African American infants [24,25].

Antenatal steroid use has been recommended for pregnancies 24 to 34 weeks gestation with threatened premature delivery to decrease the risk of RDS and neonatal death in premature neonates. ANS (dexamethasone) has been reported to be associated with decreased incidence of severe ROP [26]. However, Smith et al. have reported that single or multiple courses of ANS were not protective for the development of severe ROP [27,28]. In our study neither single nor multiple courses of ANS imparted a significant difference for the incidence of severe ROP.

In retrospective studies, postnatal steroid administration has been shown to either have a protective effect on the incidence of severity of ROP (case-control study with 58 newborns) [13], no effect (retrospective review with 147 newborns) [15], or on the contrary, increases the severity of ROP and need for cryotherapy (case control study with 52 newborns) [14].

Meta-analyses of prospective, randomized, placebo-controlled trials found no effect on the incidence of severe ROP when postnatal steroids were used early (within 96 hours after birth, eight studies, 1,453 infants) or within 7–14 days of life (five studies, 247 infants) for treatment of CLD [18,19]. However, a significant increase in severe ROP was reported when PNS was used after 3 weeks of life to treat CLD among 241 VLBW infants in six randomized placebo controlled trials (late administration) [17]. This is in accordance with our findings. The Cochrane meta-analysis reported an increased risk of 1.52 (95%CI 1.09–2.12); we found an increased odds ratio of 2.91 (Table 2).

Regarding duration, the six studies in the Cochrane review on late administration showed a wide variation in the duration of the treatment (from 6 to 42 days) [17]. Duration and dose have not yet been addressed in a meta-analysis. Two observational studies have reported an effect of duration and dose. Termote et al. reported that in VLBW infants only prolonged use of postnatal hydrocortisone was associated with an increased risk for severe ROP [29]. Haroon Parupia et al. suggested that a higher cumulative dose of PNS was a risk factor for severe ROP, controlling for other risk factors [30]. Consistent with these reports, our findings have demonstrated that ≥ 2 weeks duration, which corresponds to a total of ≥ 7 mg steroid dose, was associated with an increased incidence of severe ROP (OR: 4.09, 95% CI: 1.52–11.03). This may be indicative that only a longer duration of delayed PNS use is a risk factor for severe ROP.

Limitations of our study were the long duration of the period from 1993–2000 during which significant advances have been made in the treatment protocol of VLBW infants and could have influenced our results; however, we did not see any difference in the incidence of severe ROP during the 1993 to 1996 and 1997 to 2000 periods (Figure 1). We believe that it was an advantage that this observational study focused on a single tertiary care center with little variability in practice. In particular, the ophthalmologic examinations were consistent since all neonates were seen by the same ophthalmologist for the entire duration of the study. Another limitation is, however, that we do not have information on the follow-up of these infants after 6 months or after resolution of the ROP. Also, at the beginning of our study, reports on the effects of PNS on ROP were contradictory [13-16]. This study was initiated to explore predictive factors for severe ROP, but not to test a priory hypotheses.

In our study we have included babies with birth weight <= 1500 g based on the joint statements of American Academy of Pediatrics and the American Academy of Ophthalmology guidelines for screening premature infants for retinopathy of prematurity [31,32]. Our entry criteria differs from current practice, which is to screen neonates with birth weights <= 1250 g or <= 28 weeks of gestation based on the CRYO-ROP and LIGHT-ROP studies [33]. Our study also showed a substantial lower incidence of severe ROP in infants >28 weeks of gestational age (Table 1)

In our explanatory model for ROP, after excluding non-significant effects and non-confounding variables, the incidence of severe ROP was associated only with three factors (Table 2): gestational age, duration of ventilation, and PNS treatment. These associations were intertwined (Figures 2 and 3). Infants with lower gestational age were more likely to have been ventilated for a longer duration, received late PNS treatment for a longer period and developed severe ROP. We do not know whether late PNS treatment is the risk factor or a marker of underlying illness severity. A recent study reported short gestation, prolonged ventilation, frequent apnea and surfactant use as risk factors for developing severe ROP, however, these factors again may be the marker for the severity of illness [8].

Regarding the pathogenesis of ROP, abnormal vascular growth has been attributed to low insulin-like growth factor-1 (IGF-1) levels [34]. Genetic studies have shown that IGF-1 is critical in vessel development [35], and IGF-1 levels are correlated with birth weight and gestational age [36-39]. The premature infants who develop ROP have lower serum levels of IGF-1 than age-matched infants without disease [40]. Low levels of IGF-1 have been suggested to predict infants who will develop ROP and further suggest that early restoration of IGF-1 in preterm infants to normal levels could prevent this disease [34]. In order to achieve a better understanding of the pathogenesis of ROP, future studies should investigate the role of biologic markers. These studies can also determine the impact of risk factors such as steroids (antenatal and postnatal), gestational age, birthweight, oxygen administration, and duration of ventilation on biological markers and specific mechanisms in the pathogenesis of ROP.

## Conclusion

Prolonged use or higher cumulative dose of postnatal hydrocortisone administered to treat chronic lung disease after 3 weeks of life was associated with an increased relative risk for retinopathy of prematurity in very low birth weight neonates.

## Abbreviations

ROP, retinopathy of prematurity; ANS, antenatal steroids; PNS, postnatal steroids; NICU, neonatal intensive care unit; OR, odds ratio; 95% CI, 95% confidence intervals; ELBW, extremely low birth weight; RDS, respiratory distress syndrome; CLD, chronic lung disease; IGF-1, insulin-like growth factor-1.

## Competing interests

The author(s) declare that they have not competing interests.

## Authors' contributions

PK developed and designed the study and helped write the report. JM assisted in analyzing the data, literature review, and writing the discussion. LA conducted the ophthalmologic examinations in the infants and contributed to the interpretation. WK conducted the statistical analysis and worked on the report.

## Pre-publication history

The pre-publication history for this paper can be accessed here:


